# Practice change in chronic conditions care: an appraisal of theories

**DOI:** 10.1186/s12913-017-2102-x

**Published:** 2017-02-28

**Authors:** Melanie Harris, Sharon J. Lawn, Andrea Morello, Malcolm W. Battersby, Julie Ratcliffe, R. Doug McEvoy, Jennifer J. Tieman

**Affiliations:** 1 Flinders Human Behaviour & Health Research Unit, School of Medicine, Flinders University, Adelaide, SA Australia; 2 Health Economics Unit, Flinders Health Care and Workforce Innovations, School of Medicine, Flinders University, Adelaide, SA Australia; 3 Flinders Southern Adelaide Clinical School, School of Medicine, Flinders University, Adelaide, SA Australia; 4 Palliative & Supportive Services, School of Health Sciences, Flinders University, Adelaide, SA Australia

**Keywords:** Practice change, Chronic conditions, Theories

## Abstract

**Background:**

Management of chronic conditions can be complex and burdensome for patients and complex and costly for health systems. Outcomes could be improved and costs reduced if proven clinical interventions were better implemented, but the complexity of chronic care services appears to make clinical change particularly challenging. Explicit use of theories may improve the success of clinical change in this area of care provision. Whilst theories to support implementation of practice change are apparent in the broad healthcare arena, the most applicable theories for the complexities of practice change in chronic care have not yet been identified.

**Methods:**

We developed criteria to review the usefulness of change implementation theories for informing chronic care management and applied them to an existing list of theories used more widely in healthcare.

**Results:**

Criteria related to the following characteristics of chronic care: breadth of the field; multi-disciplinarity; micro, meso and macro program levels; need for field-specific research on implementation requirements; and need for measurement. Six theories met the criteria to the greatest extent: the Consolidate Framework for Implementation Research; Normalization Process Theory and its extension General Theory of Implementation; two versions of the Promoting Action on Research Implementation in Health Services framework and Sticky Knowledge. None fully met all criteria. Involvement of several care provision organizations and groups, involvement of patients and carers, and policy level change are not well covered by most theories. However, adaptation may be possible to include multiple groups including patients and carers, and separate theories may be needed on policy change. Ways of qualitatively assessing theory constructs are available but quantitative measures are currently partial and under development for all theories.

**Conclusions:**

Theoretical bases are available to structure clinical change research in chronic condition care. Theories will however need to be adapted and supplemented to account for the particular features of care in this field, particularly in relation to involvement of multiple organizations and groups, including patients, and in relation to policy influence. Quantitative measurement of theory constructs may present difficulties.

## Background

Chronic disease prevalence and years lived with morbidity are increasing as populations age and obesity increases [1] so that chronic conditions now account for a large proportion of health service use and costs. The Chronic Care Model is a pervasive and internationally recognised evidence-based framework for delivering an integrated approach to chronic care and articulates the general elements for improving care in health systems at the community, organization, practice and patient levels [2, 3]. These elements are: the community; the health system; self-management support; delivery system design; decision support; and clinical information systems. The Chronic Care Model has been used widely to inform service delivery across a diversity of healthcare settings, chronic condition specific contexts, and populations [4]. However, current health care systems and practices do not reflect current knowledge on minimising the burden and costs and improving outcomes for patients with these conditions [5, 6]. Bringing care delivery into line with current best practice is difficult and often unsuccessful [7] and there are many examples of clinical change failures in care for chronic conditions [5, 8–12]. In fact, failures to align service delivery with acknowledged best practice appear to be especially common in this type of care [13–15].

It is likely that the distinct and often complex characteristics of chronic care are at the root of high rates of practice change failure. Recent reviews [16, 17] clarify these characteristics and are based on comprehensive literature searches using chronic care terms such as ‘service coordination’, ‘chronic disease management’, ‘coordinated care’, ‘integrated care’, ‘cooperation’ and ‘collaboration’. For example, Valentijn et al. [16] argue that health care for chronic and overlapping health problems must be better integrated across the continuum. At the macro (system) level, the need for structures, processes and techniques which centre on the needs of the person is threatened by disease focused specialisation but also by horizontal fragmentation, for example between different primary care services. Both vertical integration (across primary, secondary and tertiary care) and horizontal integration (within primary, secondary or tertiary care) are therefore needed in the form of partnerships across traditional organisational and professional boundaries. At the meso (organisational) level, integration by different types of service organisations is required for linked-up services for a population. However, the spectrum of players, varying norms and organisational characteristics, and bureaucratic and funding structures threaten meso-level integration of care. Also included at the meso-level is the requirement to integrate care across traditional professional and disciplinary boundaries. At the micro (clinical) level, coordination is needed to achieve person-focused care, overcoming professional, institutional and sector-based boundaries and the disease focus of most interventions and guidelines. Generally, linked financial, management and information systems and supportive professional behaviour and attitudes facilitate successful integration.

Ehrlich et al. [17] similarly characterise care for people with chronic conditions as complex and requiring coordination of multiple interventions from a variety of services. They also conceptualise this care by level: the system level, service provision level and client level. At the system level, social and medical services coordination and cost-effective delivery requires resource mobilisation, information management, and organisational integration and collaboration. At the health care team level, structured frameworks, cooperative multidisciplinary teams including care coordinators, communicative and learning environments and supportive delivery systems were identified. At the client service level, coordinated care matched patient needs (using population and patient assessments) perspectives and skills and included the patient and support people in goal setting, problem-solving and provided information and support. Care plans, monitoring and review and self-management support and education were seen as important linking practices. These reviews conceptualise chronic care as complex and show the complicated and siloed care practices within and between health care, social services and related supports. This often leads to disjointed and partial service access. This, in turn, increases burdens for patients and compromises treatment linkages and therefore effectiveness [18, 19].

These complexities have been articulated recently within the holistic framework of Minimally Disruptive Medicine (MDM) [20, 21] which appears to align with and deepen current understanding of issues for chronic condition management and patient self-management and sees chronic care systems as complex and adaptive [21]. May et al. have argued that, “Chronic disease is the great epidemic of our times, but the strategies we have developed to manage it have created a growing burden for patients. This treatment burden induces poor adherence, wasted resources, and poor outcomes. Against this background, we call for minimally disruptive medicine that seeks to tailor treatment regimens to the realities of the daily lives of patients. Such an approach could greatly improve the care and quality of life for patients” ([20], p.339). An MDM approach argues that the role of patients with chronic conditions in their own care is often unrecognized or underappreciated by healthcare services and should be ‘normalized’ alongside other aspects of care through, “an awareness of the work that multimorbid patients and their caregivers must do, an understanding of the need for this work to be embedded in daily routines (and of how life circumstances can interfere with this process), and the implementation of strategies that will make care more workable to the life and context of these individuals” ([21], p.52). It is therefore, “a theory-based, patient-centered, and context-sensitive approach to care that focuses on achieving patient goals for life and health while imposing the smallest possible treatment burden on patients’ lives” ([21], p.50).

In light of the above, the structural complexities articulated by the various Implementation Science theorists will need to be addressed for successful practice change efforts that are designed to improve the integration of care across systems and stakeholders in this field. Clearly, clinical change in chronic care requires active involvement by various care delivery groups and care recipients. Care processes rely on coordinated action by individuals and multidisciplinary teams in more than one organisation and potentially across a larger system. Successful change would therefore begin with assessment of all communication and action implications within and between micro, meso and macro health service levels so all relevant groups are involved and contributing. Of central importance, chronic care also relies on the frequent involvement of patients and carers in planning and coordinating their own care, which implies action by or with patients.

Pointers from the relatively new field of implementation science can potentially improve the success of practice change in chronic complex care by helping us to understand how the many aspects of a system operate in order to achieve an integrated system. Within this field there is now a strong call for theoretical assumptions underpinning implementation research projects to be explicit [22–24]. Clarifying the concepts, definitions, and relationships that explain proposed change mechanisms can lead to interventions that are likely to succeed [24]. Also, projects with clear theoretical bases can contribute to generalizable knowledge building by providing a common structure for evaluation of potential reasons for success or failure that is underpinned by concepts that have been rigorously researched and debated, and fine-tuned over time. Additionally, with use in various implementation studies, the theories themselves are tested and can be refined towards optimising the utility of the theory base [22–24]. Terminology can however be confusing. The term ‘theory’ can be used broadly, to cover any proposed systems of concepts to account for observed phenomena [24]. However, the same term can also be used more narrowly for those systems of concepts with clear and predictive relationships between the concepts, and a degree of generalizability [25, 26]. Other terms include ‘frameworks’, where relationships between concepts may be less defined, and ‘models’, where conceptual relationships may have narrower applicability [25, 26]. Other groupings are based on the purpose and scope of the system of concepts [24, 25, 27], but these groupings overlap and may be unrelated to practical usefulness for particular implementation efforts [25]. For the work described below, we use the term ‘theory’ broadly, and inclusively.

While theoretical bases are advocated for implementation projects, there are large numbers of them and no obvious way to select the most pertinent theories for particular situations [23, 25]. In particular, available theories do not appear to have been assessed for applicability and utility in chronic care where service provision by multiple providers and multiple levels of care is a core feature, and where care is often complex due to multimorbidity or other factors. We therefore developed criteria to assess for implementation theories for this field. We then systematically appraised published implementation theories against these criteria.

## Methods

### Criteria for implementation theories relevant to care for chronic conditions

The first three authors undertook a rapid search to find reviews written within the past 10 years that reviewed the characteristics of chronic care. They considered these reviews independently and then met to discuss and agree on a tentative list of key concepts highlighted across the reviews. Through this process, the reviews led by Valentijn and Ehrlich proved particularly useful [16, 17]. Ehrlich et al. undertook a conceptual review of the literature relating to coordinated care in chronic disease in primary care settings. Their specific goal was to investigate the attributes of coordinated care which was clearly aligned with the purpose of the current analysis [16]. Valentijn et al’s work also aligned strongly with the purpose of the current analysis. Their goal was to understand the complexity of integrated care better, through the development of a conceptual framework that combined the concepts of primary care and integrated care [17]. Again, there was strong agreement by the research team that these concepts aligned well with the investigation of chronic condition care.

This tentative list of characteristics of chronic care was then presented to the larger research group of authors who each had longstanding experience and expertise in research and practice in this field. Together, the group discussed any nuances in order to reach consensus on a final list of criteria deemed important for implementation theories for this field.

### Appraisal of published implementation theories against criteria for chronic care

Lists of implementation theories have already been compiled from systematic searches of the international literature [27, 28]. The first three authors met several times to discuss and trial the use of each of these international reviews and, based their decision about how to proceed on group consensus about which review would be most straightforward to apply to this analysis. We therefore began with a recently published list of theories published from 2004 to May 2013 by Moullin et al. [27], focusing on theories applying to healthcare services and providing numerous analyses of characteristics of the listed theories. As a first step, those criteria which were partly or wholly dealt with in analyses already reported by Moullin et al. [27] were applied. The resulting list of potentially applicable theories was further assessed against remaining criteria.

The authors then met to establish a plan for assessing the identified list of implementation theories against the agreed criteria deemed necessary for care for chronic conditions. This included a detailed discussion of how the requirements could be operationalised as concise statements in order to clarify and ensure assessments were consistent across their assessments. An agreed running sheet to record assessment comments and reasoning for assessors’ choices was developed. This was pilot tested to finalise and revise the template. The first and last authors then independently assessed the theories and then met approximately one month later to discuss and workshop what they had found and agree on a final list of included theories. These were then re-presented to the larger group for discussion and finalisation.

## Results

### Criteria for implementation theories relevant to care for chronic conditions

Criteria drawn directly from reviews of chronic care [16, 17] are: applicability to change which involves individuals and multidisciplinary teams in more than one organisation, applicability at micro, meso and macro levels, and applicability to change which involves patients and carers as partners in care planning and delivery. Knowledge on the requirements to produce change in chronic care is acknowledged to be patchy [29, 30] therefore theories which allow for investigation of change requirements are needed. Prescriptive theories supplying defined action steps on the other hand appear premature and may impose an unhelpful linearity [25]. A further criterion was therefore that the theory provided structure for investigation of change requirements rather than prescribed steps. As well, so that theories can be fully assessed for this new field, a further criterion was transparent explanation of the empirical or theoretical basis of the theory. Lastly, measurement of components of theories is needed as a basis for action and evaluation across varied settings [31], therefore a further criterion was direction on structured measurement of theory components. These criteria are summarised in Table 1.

**Table 1:** Criteria to assess implementation theories for use in chronic and complex care

Criteria for implementation theories derived directly from concepts of care for chronic conditions
• Applicable generally in health care - or specifically designed for chronic and complex conditions
• Consistent with multi-disciplinary health care
• Consistent with different degrees of involvement by several organisations
• Structures assessment, action and evaluation across levels ie micro, meso and macro, ideally all three.
• Consistent with patient and potentially carer involvement in care
Broader criteria for implementation theories for chronic and complex conditions
• Provides structure for investigation of change requirements
• Transparent empirical or theoretical basis
• Provides guidance for measurements of concepts making up the theory.

### Appraisal of published implementation theories against criteria for chronic care

#### First assessment: screen using criteria of Moullin et al.

Theories identified in the systematic review of Moullin et al. [27] formed a starting list. Some of the analyses performed by Moullin et al. [27] were equivalent or partly equivalent to criteria for this study as detailed in Table 2. These were therefore used as the basis of the first assessment.

**Table 2:** Assessments using analyses of Moullin et al. [27]

Study criterion	Equivalent analysis in Moullin [27]
Theory is consistent with multi-disciplinary health care	*Orientation*, *users* not limited to one or small number of health professions
Theory applicable generally for health care - or specifically designed for chronic conditions	*Orientation* is not specific to different, narrow field
Theory structures assessment, action and evaluation across micro meso and macro levels	Related to*: Domains* include at least 2 of “individual”, “organisation” and “external system”
Theory provides structure for investigation of change requirements	Covers both *“installation”* (preparation prior to use) and *“operation”* (in use and being integrated into routine practice through active and planned approaches) of a clinical change and *Type* of framework not “prescriptive”.
Guidance is provided for measurements of concepts making up the theory	Related to: “*Elements”* include “*factors*” with definitions

Theories meeting these criteria are listed in Table 3.

**Table 3:** Theories meeting first assessment using analyses from Moullin et al. [22]^a^

Name of theory	Authors/date
Practical Robust Implementation and Sustainability Model (PRISM) This framework, developed by Canadian researchers, looks at how intervention design, external environment, organizational characteristics and the intended population influence intervention effectiveness when implementing evidence-based practices. It is based on the Chronic Care Model, the Model for Improvement and the RE-AIM framework and is targeted to all levels of staff across an organisation.	Feldstein & Glasgow 2008 [37]
Consolidated Framework for Implementation Research (CFIR) This framework, developed by US researchers focused on the health and systems of care for war veterans, was informed by Rogers’ Diffusion of Innovations Theory and the work of Greenhalgh and colleagues. The CFIR provides a menu of constructs that can be used to systematically assess potential barriers and facilitators to implementing an innovation, and provides theory-based constructs for developing context-specific logic models.	Damschroder et al. 2009 [32]
Normalisation Process Theory (NPT) This theory, developed in the UK, is an Action Theory concerned with explaining what people do individually and collectively, rather than their attitudes or beliefs, in order to ‘normalize’ complex interventions into routine practice. It contains four constructs (with each containing 4 sub-components), each representing a mechanism of social action, which is assessed against observation of what people do to implement complex interventions. The four constructs are: • Coherence - the sense-making work that people do individually and collectively when they are faced with the problem of operationalizing some set of practices. • Cognitive Participation - the relational work that people do to build and sustain a community of practice around a new technology or complex intervention. • Collective Action - the operational work that people do to enact a set of practices, whether these represent a new technology or complex healthcare intervention. Like all NPT constructs, it has four components. • Reflexive Monitoring - the appraisal work that people do to assess and understand the ways that a new set of practices affect them and others around them.	May et al. 2009 [33]
General Theory of Implementation This theory builds on NPT, informed by ideas about agency and its expression within social, social and cognitive mechanisms, and collective action. It incorporates these ideas from sociology and psychology to build a more comprehensive explanation of change. That is, it acknowledges that change occurs within a social system involving context and emergent expressions of agency. Context includes potential (individual and collective commitment) and capacity (material and cognitive resources, social roles, social norms). Emergent expressions of agency includes capability (workability and integration) and contribution (which include the original 4 constructs of NPT)	May et al. 2013 [34]
Promoting Action on Research Implementation in Health Services (PARiHS) This framework, developed by researchers in Australia and the UK, examines the interactions between three key elements for knowledge translation: evidence; context; and facilitation. It argues that successful implementation of evidence into practice had as much to do with the context or setting and how that new evidence was introduced as it had to do with the quality of the evidence. It incorporates themes from the organisation change literature such as planning, knowledge and skills. Each element consists of sub-elements that can be rated on a scale from low to high.	Kitson et al. 2008 [26]
Revised PARiHS framework for a task-oriented approach to implementation This framework, developed by researchers in the US independently of the original developers, is designed to enable users to more clearly and consistently define and apply relevant terms with the PARiHS. It aims to address: the lack of conceptual clarity, specificity, and transparency; the lack of inclusion of relevant elements perceived to be critical to implementation; and the lack of instrumentation and evaluation measures in the original framework.	Stetler et al. 2011 [35]
Critical Realism and the Arts Research Utilization Model (CRARUM) This model, developed by researchers in Canada, draws on Critical Realism to provide insight into the interrelationship between its structures and potentials, and individual action and the Arts to foster reflection on the ways in which context influences and shapes clinical practice, and how they may facilitate or impede change. It draws on The Ottawa Model of Research Use which considers a range of factors across the assessment, monitoring and evaluation continuum. In particular is stresses the importance of understanding the optimization of intervention and adoption strategies, including an assessment of the knowledge, attitudes and skills of potential adopters.	Kontos & Poland 2009 [38]
Sticky Knowledge This model, developed by researchers in the UK, is based on an integration of communication theory and knowledge transfer milestones in a primary care context. The researchers argue that knowledge factors play a greater role in the success or failure of a knowledge transfer than has been suspected. The model’s key knowledge factors (Predictors of stickiness at different points of knowledge transfer) include: causal ambiguity, unproven knowledge, motivation of source, credibility of source, recipient motivation, recipient absorptive capacity, recipient retentive capacity, barren organizational context, and arduous relationship between source and recipient.	Elwyn, Taubert & Kowalczuk 2007 [36]
Advancing Research and Clinical Practice Through Close Collaboration Model This model, developed by researchers in the US, stresses that the key strategy to sustain evidence-based care is the presence of an evidence-based practice (EBP) mentor (a clinician with advanced knowledge of EBP, mentorship, and individual as well as organizational change). In involves an assessment of organizational culture and readiness for EBP that includes an assessment of potential strengths and barriers, followed by the development of mentors as a core feature of then implementing strategies to build skills, assess and address beliefs about EBP, and evaluate EBP implementation.	Melnyk et al. 2010 [39]

^a^Our judgment differed from that of Moullin et al. [27] in relation to three theories. We judged that “installation” was covered by *General Theory of Implementation*, and that “setting” was not limited to hospital and primary care for *PARIHS* and *Sticky Knowledge* respectively. We therefore included these theories above. Moullin at al [27] list a further *PARIHS* theory [43]. We excluded this because in our judgement the referenced publication did not provide a theory

#### Second assessment; application of further criteria for chronic care

Remaining requirements were operationalised as concise statements so that judgments could be made about whether or not a theory complied with this requirement. The resulting criteria are shown in Table 4.

**Table 4:** Criteria and operation

Study criterion	Application
Theory structures assessment, action and evaluation across micro meso and macro levels	Explicit coverage of patient care, service and policy levels
Theory allows for active involvement by more than one organisation	Theory explicitly or structurally allows for involvement of several organisations in creating a change
Theory allows for active involvement of patients can carers	Theory explicitly or structurally allows for involvement of patients and carers in creating a change
Empirical or theoretical bases explicit	Each theory component supported by linked references, including structured reviews or theoretical arguments.
Guidance is provided for measurements of concepts making up the theory	Measures are described

Further analyses against these remaining criteria are shown in Table 5. Six theories partially or wholly met at least four of the five criteria. These were CFIR [32], NPT [33], General Theory of Implementation [34], PARIHS [26], the PARIHS variant of the Veterans Health Administration of the United States of America (USA VHA) [35], and Sticky Knowledge [36].

**Table 5:** Analysis of preliminary list against features of change chronic and complex care

Theory^a^	Relevant to micro, meso and macro levels	Clearly allows for active involvement by more than one organisation	Patient involvement	Empirical or theoretical bases explicit	Practical measurement tools in key reference?
PRISM Feldstein & Glasgow 2008 [37]	Partly – “macro” barely covered	No	Yes	No - Basis for selecting references not given	Yes
**CFIR Damschroder2009** [32]	Partly – “macro” barely covered	No	Somewhat – seeing patients as targets	Yes	Yes Comprehensive discussion and referencing of aspects to be measured
**NPT May 2009** [33]^a^	Partly – “macro” not covered	Consistent but not explicit	Consistent but not explicit	Yes	Partly – Broad outline presented for measurements
**General theory of implementation May 2013** [34]	Partly – “macro” not covered	Consistent but not explicit	Consistent but not explicit	Yes	Partly - Measurement conceptually explained
**PARIHS Kitson et al. 2008** [26]	Partly – “macro” not covered	Consistent but not explicit	Addressed in evidence – not as active in implementation.	Yes	Yes - Guide for qualitative data collection.
**Revised PARIHS framework for USA VHA Stetler 2011** [35]	Partly – “macro” not covered	No	Somewhat – seeing patients as targets	Yes	Yes – Measures explicitly outlined
CRARUM Kontos 2009 [38]	Partly – “macro” not covered	No	No	Yes	No
ARCC Melnyk 2010 [39]	No – micro barely covered, focus of meso is nursing and macro absent	No	No	No	Partly - 2 proposed scales are referenced
**Sticky knowledge Elwyn 2007** [36]	Partly – “macro” not covered	Consistent but not explicit	Yes	Partial – describes and references one theory from another discipline	No – Little basis provided for measurement.

^a^Theories named in bold are those wholly or partly meeting at least 4 of the 5 criteria

No theory uniformly covered all levels of the health system, macro meso and micro. Policy (macro) level change in particular was either barely treated or absent in all the examined theories. In addition, no theory clearly dealt with the involvement of several organisations in a clinical change. Some theories however were described in a way that appeared applicable for multiple organisational settings. These included NPT [33] and the linked General Theory of Implementation [34], PARIHS [26] and Sticky Knowledge [36]. Others appeared specifically designed for use by single organisations, indicated by use of terms such as “target site” [31] and “the organization” [32, 37]. Only PRISM [37] explicitly included patients as active contributors to clinical change. Some other theories however were described generically in a way that appeared applicable for multiple contributing groups, including patients. These included NPT 2009 [33] and the linked General Theory of Implementation [34] and Sticky Knowledge [36]. Other theories either positioned patients as recipients of changes determined by others [26, 32, 35] or essentially disregarded any patient role [38, 39]. Clear empirical and theoretical bases were provided for most theories. However, for two the bases were less transparent [37] or essentially absent [39]. Most theories provided definitions or links to tools for factors as a basis for measurement [26, 32–35, 37, 39] but two did not [36, 38].

## Discussion

Given the large and increasing health care and personal burden associated with chronic conditions, better application of research evidence is critical. Identifying and using theoretically-grounded approaches is the recommended starting point for efforts to better apply research evidence in health care practice. This review therefore sought to identify theories (and similar systems of ideas) that address the particular characteristics of implementation of change in chronic care. While no implementation theory was judged to meet all criteria to a high level, six met most criteria at least partially and have potential in structuring research in clinical change in chronic care. These were CFIR [32], NPT [33] and its extension as General Theory of Implementation [34], PARIHS [26] and the variant of PARIHS developed for the USA VHA [35], and Sticky Knowledge [36]. Each has particular characteristics which may be relevant for particular implementation settings and purposes.

The identified theories differ in their origins, and in the development of measurement tools. The NPT is a transparently developed middle range sociological theory with well-defined components. The focus of NPT is on the social context and requirements for practice change [34]. Quantitative measurement tools are being created for NPT by the theory developers [40–42]. The broader General Theory of Implementation includes NPT and definitions of additional concepts are provided as a starting point for development of measures [34]. The PARIHS framework [26] was proposed from inductive analyses of implementation cases. It is presented as a framework with development work still needed, including on included concepts, definitions, and measurement [26, 43]. The variant of PARIHS created by the USA VHA [35], however, includes detailed concept definitions and questions to structure qualitative assessment of the concepts. Some quantitative measurement tools have also been developed and used by other groups [44, 45] though mostly in single-setting changes. The CFIR is a synthesis and re-definition of constructs from pre-existing theories rather than a completely new theory [32]. Quantitative scoring of CFIR components from qualitative interviews has been described [46] but validated measures specific for this framework are still lacking [47]. Sticky knowledge is a theory from the field of business management, not well known in health but with potential application described in the key paper [36]. Measurement tools, however, are oriented to business applications [48].

The identified theories have also been used to different extents in practical implementation projects. Structuring the analysis of retrospectively collected qualitative data appears to be the main reported use of theories, generally [49, 50] and in chronic and complex care [9]. Prospective use and testing of theories therefore remains an urgent requirement [50].

There are indications that theories identified in this review may need to be modified [43]. For example, while some components of the USA VHA version of the PARIHS framework were positively associated with degree implementation in a mental health service change, other components were negatively associated [51]. A study using the original PARIHS framework [43] found that it did not adequately cover the influence of practitioners and patients actions and recommended extension to capture the influence of individuals. In a study using the CFIR to evaluate care transition interventions, adaptations were required to broaden the scope of the settings concept to organisations and to bring in patient- and caregiver-centeredness [52]. A review of the NPT [49] conducted by members of the NPT development team identified some apparent overlap between constructs but few suggestions for expansion. Of course, this theory testing can only occur when theories are used in research projects. Less used theories such as Sticky Knowledge are therefore still to be assessed in health service practice [36].

Unfortunately, while policy-level action is particularly important in chronic and complex care, [48] none of the identified theories thoroughly addresses implementation requirements at the policy level. Implementation theories generally characterise policy processes as messy and unpredictable but provide little guidance [53, 54]; therefore, separate theories are needed for this aspect. Broad policy-use theories [55] and theories from related fields [56] may be applicable, and a specific health policy research framework [54] has recently been proposed which submits that use of research in policy requires a catalyst and capacity within the policy organisation and that, if sufficient capacity is present, research engagement actions might facilitate research use. One could readily imagine using elements of NPT or PARIHS in policy contexts, though their use might arguably render the object of analysis to again focus on the meso-organizational level, at the expense of the need to also focus on and integrate the macro and micro levels sufficiently.

A further consideration to address implementation requirements at the policy level could be to theory aligned with a Health in All Policies (HiAP) approach [57]. HiAP acknowledges that health is an outcome of all policies and requires multi-sectoral action, underpinned by health equity. It is underpinned by the principles of legitimacy grounded in rights and obligations, accountability of governments towards their community, transparency, participation of the wider community in policy development and implementation, sustainability of policy to meet the needs of current and future populations, and collaboration across sectors. HiAP “improves accountability of policymakers for health impacts at all levels of policy-making. It includes an emphasis on the consequences of public policies on health systems, determinants of health, and well-being” [57]. It appears to align well with considerations about chronic condition care and supporting self-management by people with chronic conditions which emphasize the importance of multiple psychosocial factors within the person’s context that contribute the effective management of their chronic conditions.

In addition, only one theory (PRISM) [37] explicitly included patients as active contributors to clinical change, and none of the six theories explicitly incorporates the idea of active involvement of self-managing patients and carers as part of that change. However selected theories, notably NPT, General theory of implementation and sticky knowledge appear consistent with the inclusion of patients and carers as part of care activities and therefore change initiatives. This is a significant gap in existing theories given the pervasive focus on chronic condition self-management by the patient and self-management support by healthcare providers, carers and other support providers as a central element of chronic care. Chronic conditions are long-term, often requiring daily management over several years with the potential to have significant negative impacts on physical, social and emotional wellbeing. The needs of patients with chronic conditions are therefore likely to be pervasive throughout health and social care systems over extended periods of time. Therefore, any theories designed to support the implementation of change in healthcare systems must capture the complexity of vertical and horizontal layers that seem so important for effective chronic care. The holistic framework of MDM [20], as already stated, stresses the realities of patients’ lives, which suggests the need for theory that truly puts the person with chronic conditions at the centre, given the potential complexities of need when living with a chronic condition.

The need for prospective studies to be based on explicit theories has been highlighted. Researchers and health professionals in clinical change research should also justify their choice of particular theories, for example in selecting one of the theories identified in this review. Rationales for selection of particular theories or theory-based change strategies are currently rarely explicit in published research [58]. Some guidance is available on how to choose between candidate theories. For example, Wensing et al. [59] discuss opinion based and theory-based methods, and Powell et al. [60] describe a range of formal processes including concept mapping, group model building, conjoint analysis, and intervention mapping. Analyses presented in this study can now contribute to use of such processes for theory selection in research into clinical change in chronic care by identifying strengths and gaps that exist in the available implementation theories when applied to this important and growing area of healthcare.

## Conclusions

Overall, systematic knowledge building is necessary to underpin more successful implementation of change in chronic care. Using theories to guide and evaluate change strategies is likely to lead to more generalizable findings. This review identifies six theories that may have particular value in structuring clinical change in chronic and complex care, but theories may need to be adapted and valid measures developed for the concepts making up these theories.

## Abbreviations

CFIR: Consolidated Framework for Implementation Research
CRARUM: Critical Realism and the Arts Research Utilization Model
MDM: Minimally Disruptive Medicine
NPT: Normalization Process Theory
PARIHS: Promoting Action on Research Implementation in Health Services
PRISM: Practical Robust Implementation and Sustainability Model
USA VHA: United States of America, Veterans Health Administration
